# Intravenous Tranexamic acid versus placebo during Caesarian section: A comparative study

**DOI:** 10.12669/pjms.38.5.5383

**Published:** 2022

**Authors:** Muhammad Jawad Iqbal, Atifa Mazhar, Alina Shabir

**Affiliations:** 1 Dr. Muhammad Jawad Iqbal, MBBS, MO, Department of Gynecology. Nishtar Medical University & Hospital, Multan, Pakistan; 2 Dr. Atifa Mazhar, MBBS, MO, Department of Gynecology. Nishtar Medical University & Hospital, Multan, Pakistan; 3 Dr. Alina Shabir, MBBS, MO, Department of Gynecology. Nishtar Medical University & Hospital, Multan, Pakistan

**Keywords:** Tranexamic acid, post-partum hemorrhage, Cesarean section

## Abstract

**Objectives:**

To evaluate the effectiveness of Tranexamic Acid in preventing postpartum hemorrhage against placebo in high-risk women undergoing cesarean section.

**Methods:**

A double-blinded placebo-controlled comparative trial was conducted at the Obstetrics and Gynecology Department of Nishtar Hospital for one year, from 15^th^ June 2020 to 15^th^ June 2021. A total of 60 women who were at high risk of postpartum hemorrhage and had to undergo elective cesarean sections were included in the study. Among them, initial 30 patients were administered Tranexamic Acid before skin incision whereas later 30 were treated as the placebo group. These women were then observed for blood loss during surgery and within 24 hrs. after surgery and any postoperative complications such as thromboembolic events, the need for additional uterotonic agents, and blood transfusions.

**Results:**

Out of 60 women, 30 were placed in each group. The groups had no significant difference in terms of baseline data and post-partum hemorrhage-associated risk factors (p>0.05). However, the occurrence rate of primary post-partum hemorrhage (blood loss greater than 1000 ml) was significantly less in a tranexamic acid group than the placebo group (16.6% vs 60%, respectively, p<0.01). Similarly, the requirement of additional uterotonic agents (13.3% vs 43.3%, respectively) and the need for blood transfusion (6.0% vs 23.3%, respectively) was lower in a tranexamic group than in the placebo group.

**Conclusion:**

The study highlighted the significance of tranexamic acid in controlling post-partum hemorrhages, the requirement of additional uterotonic agents, improving post-partum hemoglobin, and the need for blood transfusion.

## INTRODUCTION

Postpartum hemorrhage (PPH) is one of the major causes of maternal mortality and morbidity across the world.^1^ Around 25% of maternal deaths are accounted for PPH and about 12% of survivors of PPH report severe postpartum anemia.^2^ Although in the last decade, remarkable improvement has been witnessed in the PPH-related mortality scores, the developing countries, like Pakistan, continue to suffer from high PPH and its consequences.^3^

The majority of PPH-associated maternal complications and deaths could be minimized or prevented with on timely provision of emergency care and effective treatment.^4^,^5^ Although most of the PPH-affected women are found to be associated with no major historical predominance or clinical risk factors, researchers tend to relate multiparity with a high risk of postpartum bleeding.^6^,^7^ Generally, in around 75% of PPH cases, Uterine Atony causes bleeding during or after caesarian. However, it only leads to maternal mortality in about 6.4% of cases while other conditions of obstetric hemorrhage such as the ruptured uterus, placenta praveia, abruption, cervical and vaginal trauma, inverted uterus, and adherent placenta mostly result in maternal death.^8^,^9^ Currently, various measures including administration of uterotonics, prostaglandins, and active management of 3^rd^ stage of labor are used to prevent PPH. According to Cochrane review, through active management of labor the incidence of PPH> 1000 ml has been significantly reduced.^7^ However, in scarce resource setup, including that of Pakistan, no prominent decline in PPH-associated maternal death rate could be achieved, despite extensive use of uterotonic. Thus, the issue requires advanced interventions to be addressed effectively.

Tranexamic acid (TA) is considered effective agents against blood loss and has been extensively utilized in diverse medical complications, such as hemorrhage, intra and postoperative blood loss.^10^ A study conducted by World Maternal Antifibrinolytic (WOMAN) claimed that tranexamic acid not only reduces the PPH-associated death rate, from 1.9% to 1.5% but also has no side effects.^6^ Hemorrhage (CRASH-2) study reported that early prophylactic administration of tranexamic acid reduces bleeding severity, demand for additional uterotonic agents, and related maternal mortality.^11^

Regardless of the extensive use of various uterotonics, PPH associated mortality rate seems to remain constant, or even found to be increasing, in developing states such as Pakistan. Moreover, in Pakistan, not enough larger studies have been conducted to assess the efficacy of tranexamic acid. Similarly, most of the existing literature has excluded high-risk patients in the trials. This randomized, double-blinded, and placebo-controlled study was designed to assess the effect of TA in reducing bleeding during and after the elective caesarian sections (CS) in high-risk, pregnant women to provide an effective and reliable pharmacological therapy for PPH in low resource settings of Pakistan. The Trial group in our study had received TA as an additional protective measure against blood loss associated with the procedure while the placebo group was receiving all the standard care besides normal saline which was just the placebo treatment to keep the participants blinded to the type of intervention which addresses the ethical question of keeping high-risk women in this group on normal saline.

## METHODS

A randomized, placebo-controlled study was conducted at the Department of Obstetrics and Gynecology of Nishtar Hospital, for the period of one year from 15^th^ June 2020 to 15^th^ June 2021. The women were included in the study after getting confirmed for prospective elective CS, who had passed the gestational period of 38 weeks, and those who had at least one of the following risk factors: multiple gestations (>4), failed induction, obstructed or augmentation of labor, placenta praevia, chorioamnionitis, polyhydroamnious, coexisting fibroids, fetal macrosomia, pregnancy-related hypertensive disorders, and history of CS or PPH. The elective caesarian section was characterized as the CS conducted before the start of labor. Whereas, women with other coexisting comorbidities such as cardiac, liver, and renal disorders, bleeding disease with a history of anticoagulant use, and allergic reaction to TA were excluded from the study. A total of 60 women were included in the study after seeking their informed consent and getting ethical approval ref no.128/42 on dated 20/05/2020 from the hospital.

The included women were then randomly placed into two groups: the Trial group and placebo group (30 women in each group), by allowing them to covertly select envelopes marked with P or T. The allocation of the participants in two groups was only shared with the anesthesiologists as they had to administer TA and normal saline accordingly while obstetricians and patients were kept blinded to the group allocation. Accordingly, women in the Trial group were administered with 1g of TA (diluted in 10ml of 0.9% normal saline), intravenously. Whereas, women in the placebo group were given an intravenous dose of 20ml of 0.9% normal saline instead. Soon after the clamping of the umbilical cord, all the women were given an infusion of 40IU of diluted oxytocin, over three hours. The requirement of any additional uterotonic drugs during or within 24hrs after the surgery was systematically noted. Besides, any need for intra or postoperative blood transfusion (following severe intraoperative bleeding or postsurgical hemoglobin (Hb) less than 8g/dl), post-operative Hb levels up to 48 hrs. The total amount of intraoperative blood loss, or any other adverse maternal events were also recorded. In this regard, maternal blood loss (in liters) was calculated by using Nadler formula:^12^

**(0.3669 × H^3^) + (0.03219 × W) + 0.6041**

Where “W” is weight in kilograms and “H” is height in meters.

Moreover,

### Percentage of blood volume lost = (preoperative Hb/ postoperative Hb)/ preoperative Hb:

All the patients in the trial group were counseled on the possible occurrence of thromboembolic events and were explained the related signs and symptoms. All these women were followed for incidence of thromboembolic events up to six weeks.

All the relevant data, including baseline characters, PPH associated risk factor(s), and preoperative hematocrit level (recorded within 24 h before the surgery) were recorded. The requirement of any additional uterotonics (if any) within one day following delivery was regarded as the primary outcome, whereas, the secondary outcome measures included approximate blood loss, severe blood loss (more than 1000ml), and the need for blood transfusion.

All the data were statistically analyzed through SPSS (Version 19). The quantitative data were presented in the form of mean ± standard deviation (SD), while the percentage of qualitative data was computed. The categorical variables between the two groups were compared through the Chi-square test (X2) while the student’s t-test was used for the comparison of numerical variables. A P-value for less than 0.05 for any variable was considered statistically significant.

## RESULTS

A total of 60 high-risk pregnant women were included in the study such that 30 were placed randomly in each study group. The two groups were matched for their baseline characteristics and predisposing risk factors for PPH and no significant difference could be found between the two study groups (Table-I and Table-II).

**Table I:** Baseline Characteristics of the Participants in both Groups (N=60).

Variables	Trial Group (n=30)	Placebo Group (n=30)	P-Value
Age (years)	27.6 ± 4.3	27.9 ± 4.7	0.61
BMI (kg/m^2^)	24.1 ± 2.3	24.7 ± 2.7	0.22
Parity	2.0 ± 1.3	1.7 ± 1.1	0.7
Gestational age (years)	35 ± 2.7	37 ± 4.7	0.91

**Table II:** Comparison of PPH associated Risk Factors (N=60).

Variables	Trial Group, n=30 (%)	Placebo Group, n=30 (%)	P-value
Multiparity (≥4)	22 (73.3)	18 (60)	0.35
Eclampsia	7 (23.3)	9 (30)	0.78
Chorioamnionitis	3 (10)	1 (3.3)	0.65
Placenta previa	4 (13.3)	2 (6.6)	0.7
Abruption placentae	1 (3.3)	0 (0)	0.91
Polyhydramnios	0 (0)	1 (3.3)	0.9
Fetal macrosomia	12 (40)	9 (30)	0.86
Coexisting fibroids	7 (23.3)	9 (30)	0.77
Previous history of PPH	5 (16.6)	3 (10)	0.89
Oxytocin augmentation	11 (36.6)	13 (43.3)	0.92
Labor induction	17 (56.6)	20 (66.7)	0.65

The mean preoperative Hb levels in both groups were almost equivalent and no statistical difference could be found. However, 48 hour postoperative Hb level was significantly higher among women in the Trial group (10.8 ± 1.5, P-value < 0.01) in contrast to the women in the Placebo group (7.3 ± 1.1) (Table-III). Similarly, the amount of blood loss was significantly lower upon administration of tranexamic acid in the Trial group (390 ± 115 ml, P-value < 0.001) as compared to those in the Placebo group (812 ± 110 ml) (Table III). Consequently, the number of patients with severe blood loss (>1000) was also significantly less in the Trial group as to the other arm of the study (16.6% versus 60%, respectively, P-value < 0.01). The significantly lower number of patients in TA groups required blood transfusions (6.6%) and additional uterotonic (13.3) in contrast to their counterparts in the placebo group, 23.3%, and 43.3% respectively.

**Table III:** Primary and Secondary Outcomes of the Trial (N=60).

Variables	Trial Group, n=30	Placebo Group, n=30	P-value
Preoperative Hb (g/dl)	11.0 ± 1.9	12.2 ± 2.3	0.34
Postoperative Hb (g/dl)	10.8 ± 1.5	7.3 ± 1.1	<0.01
Calculated blood loss (ml)	390 ± 115	812 ± 110	<0.001
Blood loss more than 1000 ml	5 (16.6)	18 (60)	<0.01
Required transfusion	2 (6.6)	7 (23.3)	<0.05
Additional uterotonics	4 (13.3)	13 (43.3)	0.23

## DISCUSSION

This study evaluated the efficacy of TA in reducing the blood loss during and after the elective CS and found it effective in blood loss reduction against placebo (390 ± 115 ml versus 812± 110 ml, respectively). The result of this study complies with a former Turkey-based study which reported the significant role of TA in reducing blood loss level at 48 h following the surgery as compared to the placebo group (499.9 ± 206.4 ml vs. 600.7 ± 215.7 ml, respectively).^11^ Similar results were reported by related studies conducted on Indian and Egyptian women.^12^-^14^

A significant reduction in the incidence of primary postpartum hemorrhage (blood loss greater than 1000 ml) upon receiving TA was observed. In a similar study conducted by Gai et al., the researchers reported comparable results where patients in TA groups had a mean loss of 351 ml of blood during the operative procedure against 440 ml in the control group.^15^ In recent years, trials are also conducted to confirm the efficacy of TA on blood loss during vaginal delivery. In this regard, Xia and his colleagues^16^ tested the agent on women undergoing normal delivery and found it effective in reducing the incidence of PPH, and postoperative blood loss.^16^

According to the above-mentioned study, intake of TA significantly reduces the need for any additional uterotonics which goes hand in hand with the results of the previous studies.^9^,^17^ However, the higher intake of oxytocin is reported in the TA group as compared to earlier discussed Turkey-based study. This is probably due to the higher participation of high-risk women in the present study.

We have also reported a comparatively lesser requirement of post-surgical blood transfusions as compared to placebo (2 women vs 7 women, p<0.05). This finding complies with the earlier reported results in similar studies.^8^,^18^ However, Bhatia and Deshpande reported no significant difference in the blood transfusions needs between the two study groups in their study.^14^ Again, the difference in the results could be due to the selection of the study population (high-risk women in our case).

### Limitations of the study:

The study is limited in terms of its inability to report the decrease in any influence of treatment over maternal death rate or requirement of any other invasive procedure. Similarly, due to a limited follow-up period, TA-related thromboembolic events couldn’t be accurately evaluated.

## CONCLUSION

In conclusion, the study highlighted the significance of tranexamic acid in controlling post-partum hemorrhages, the requirement of additional uterotonic agents, improving post-partum hemoglobin, and the need for blood transfusion.

### Authors’ Contribution:

**MJI, AS:** Conceived, designed and did statistical analysis & editing of manuscript.

**AM, MJI:** Did data collection and manuscript writing.

**AS, AM:** Did review and final approval of manuscript.

**MJI, AS:** Responsibility of authentication of study.
